# Longitudinal Predictive Control for Vehicle-Following Collision Avoidance in Autonomous Driving Considering Distance and Acceleration Compensation

**DOI:** 10.3390/s22197395

**Published:** 2022-09-28

**Authors:** Shutong Yin, Chunlin Yang, Ibna Kawsar, Haifeng Du, Yongjun Pan

**Affiliations:** College of Mechanical and Vehicle Engineering, Chongqing University, Chongqing 400044, China

**Keywords:** vehicle following, collision avoidance, safety distance model, optimal control theory, acceleration compensation

## Abstract

In response to the widespread adoption of vehicle-following systems in autonomous applications, the demand for collision warning to enable safer functionalities is increasing. This study provides an approach for automated vehicle guidance to follow the preceding vehicles longitudinally and puts emphasis on the performance of collision avoidance. The safety distance model is established, which contains a distance compensation algorithm to deal with the special case on curved roads. By introducing the algorithm of velocity and distance prediction, the collision risks are detected and measured in real time. The objective function is established based on optimal control theory to solve the desired following acceleration. The control system designed with the method of proportion integration differentiation combines throttle percentage and brake pressure as outputs to compensate acceleration. In the Carsim and Simulink co-simulation platform, the control system for longitudinal collision avoidance is simulated and analysed for four typical working conditions: the preceding vehicle drives at a constant speed on straight and curved roads, while the preceding vehicle drives at various speeds on straight and curved roads. The results validate the feasibility and effectiveness of the proposed method, which can be used for the longitudinal control of vehicle-following active collision avoidance.

## 1. Introduction

Autonomous driving has gained great attention with rapid advancements. It is considered to be one of the most effective ways to reduce traffic accidents and enhance transportation system performance [1,2]. The design of an active collision-avoidance system is a topic of immense interest across the globe to promote driving safety [3,4,5]. In recent years, the technologies of forward collision warning (FCW) and autonomous emergency braking (AEB) have been continuously ameliorated and proven effective [6,7]. The vehicle-following concept has been universally adopted in several autonomous applications, e.g., adaptive cruise control (ACC) and platooning [8,9,10]. A multitude of vehicle designs have been developed and implemented in this context, commonly consisting of two important components: a spacing policy and an associated control system [11]. A spacing policy defines the desired value of following distance as a function of vehicle speed, while an associated control system regulates the real distance in pursuance of it.

Tang et al. proposed a vehicle-following model with consideration of the two safety distances (i.e., the front safety distance and back safety distance) [12]. To deal with the complexity of highway conditions, Fang et al. came up with a composite in combination with the constant time headway model and the driver-preview safety distance model [13]. Further considering the subjective factor of the driver, Luo et al. established a improved vehicle-following safety model based on different reaction time [14]. As to the design of the control system, Canale et al. devised a 2-DOF controller with feedforward and feedback control methods in combination [15]. The control parameters could be adjusted to different driving characteristics. Kim et al. presented a novel approach for time-varying parameter adaptive throttle and brake control for vehicle speed tracking, enjoying high robustness and generalization [16]. Focusing on an overall aim of the comprehensive improvement of safety, comfort, and economy, Zhang et al proposed a novel advanced emergency braking system (AEBS) with a multi-objective optimization controller using the theory of non-linear model predictive control [17].

**Figure 1:**
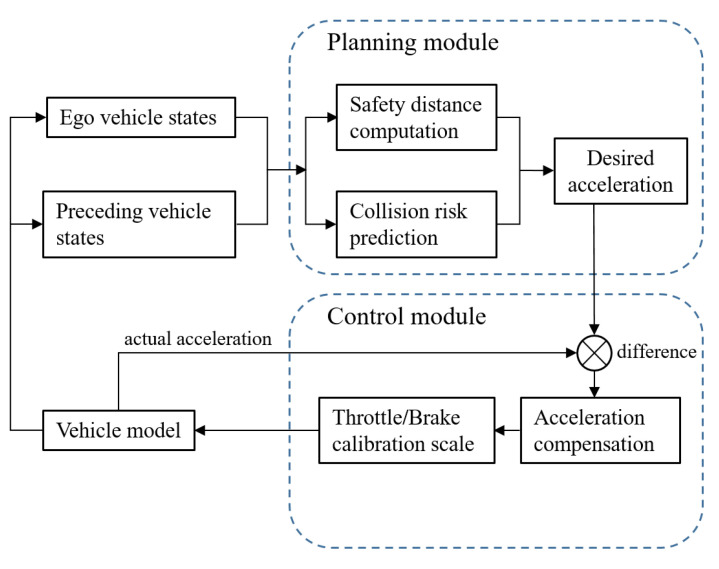
The controlling structure.

However, collision easily happens when there is a low speed or sudden deceleration in the preceding vehicles. In the absence of predictive warning, real-world applications in vehicle-following scenarios pose certain risks [18]. The need to dynamically evaluate and predict collision risks during the course is increasing to enhance the safety performance [19,20]. Motivated by this, we focus on the longitudinal control for active collision avoidance in vehicle-following scenarios. In this work, we couple the motion of the ego vehicle and the preceding vehicles. The state information (velocity, acceleration, etc.) is collected from several vehicle sensors and is further utilized to carry out the computation of safety distance and collision risk. Based on the optimal control theory, an objective function is then established to solve the desired acceleration, and a longitudinal proportion integration differentiation (PID) control algorithm is introduced for acceleration compensation. Significantly, the objective function considers both the speed cost and distance cost, offering the necessary features to cover dynamic driving situations. The model of safety following distance is embedded in the distance cost to enhance safety capability. Finally, the controlling performance is investigated under different running modes in simulation. The logic structure is shown in Figure 1.

The reminder of the manuscript is organized as follows. Section 2 depicts the model of safety following distance. Section 3 utilizes the optimal control theory to solve the desired acceleration, and in Section 4, the longitudinal PID control method is exploited for acceleration compensation. Section 5 simulates the control system and analyses the performance. Section 6 concludes the paper and discusses future opportunities.

## 2. Safety Distance Model

**Figure 2:**
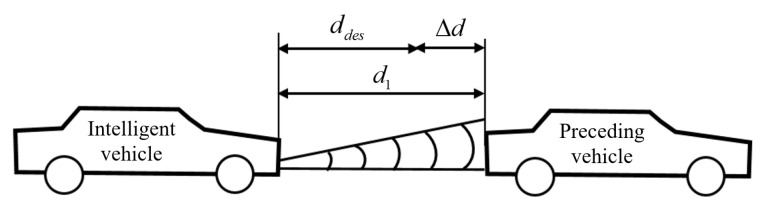
Longitudinal following on straight roads.

The depiction of following distance plays a crucial part in determining safety performance. Shorter distances enhance traffic efficiency and mobility at the expense of traffic safety (like an increase in rear-end collisions). However, longer distances are of great uncertainty as other traffic participants could interfere with the path. Time headway is an important evaluation indicator and is commonly used in depicting safety following performance. In this section, based on the relative motion between ego vehicle and preceding vehicle, a model of safety following distance is described using a fixed time headway [21]. The state of longitudinal vehicle-following on straight roads is described in Figure 2.

The ego vehicle uses sensors to detect the distance $d_{1}$ and the relative velocity Δv with preceding vehicles, based on which $d_{des}$ and Δd can be given by:

(1) $$d_{des}=t_{h}v_{1}+d_{0}$$

(2) $$\Delta d=d_{1}-d_{des}$$

where $d_{des}$ is the desired value of safety distance, $v_{1}$ is the velocity of ego vehicle, and Δd is the difference in safety distance between the desired value and actual value. $t_{h}$ is the time headway, ranging from 1.2 to 3 s according to different steering requirements. $d_{0}$ is the minimal value of safety distance, ranging from 2 to 5 m.

It shows Equations (1) and (2) that the desired safety distance is proportional to the velocity of ego vehicle and can be flexibly adjusted to accommodate diverse driving habits. However, a sharp deceleration makes it problematic for riding safety and comfort when there is a high speed in the ego vehicle but a low speed in the preceding vehicles. On this account, the influence exerted by the difference of velocity between ego and preceding vehicles should be valued. Here, a buffer built against great differences of velocity and Equation (1) can be rewritten as:

(3) $$d_{des}=t_{h}v_{1}+d_{0}-k\Delta v$$

where, $t_{h}$, $d_{0}$, and *k* have the value 1.6 s, 5 m, 0.375, respectively [22]. In view of practical necessities, by introducing the saturation function to limit the range of $d_{des}$, Equation (3) can also be rewritten as [23]:

(4) $$d_{des}=\mathrm{sat}\left(d_{des}\right)=\left\{\begin{array}{cc} d_{\max} & \;t_{h}v_{1}+d_{0}-k\Delta v>d_{\max}\\ t_{h}v_{1}+d_{0}-k\Delta v & \;d_{0}\leq t_{h}v_{1}+d_{0}-k\Delta v\leq d_{\max}\\ d_{\min} & \;t_{h}v_{1}+d_{0}-k\Delta v<d_{0} \end{array}\right.$$

where $d_{\max}$ has the value 100 m as the maximum following distance in safe situations and $d_{\min}$ has the value 5 m as the minimum when there is a short distance between ego and preceding vehicles.

However, millimeter-wave radars detect only the straight-line distance, making it problematic for vehicle following on curved roads. The system stability suffers as the judging of following target would be easily intervened if adjacent vehicles are detected closer in straight line.

**Figure 3:**
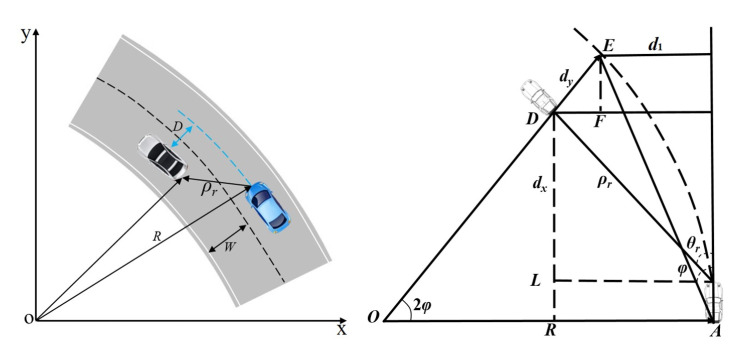
Longitudinal following on curved roads.

Here, we introduce a distance compensation algorithm supposing that the curve radius is longer than the distance radars can detect and the ego vehicle and adjacent vehicles share the same road curvature [24]. The state of longitudinal vehicle-following on curved roads is described in Figure 3. The ego vehicle is at *A*, and there is an adjacent vehicle at *D*. *E* is the vertical projection of *D* on the trajectory of the ego vehicle.

Yaw velocity can be measured by vehicle sensors, and the road curvature can be given by:

(5) $$C=\frac{\omega }{v}=\frac{1}{R}$$

where *C* is the road curvature, *v* is the velocity of ego vehicle, ω is the yaw velocity, and *R* is the curve radius.

Based on the geometry, the distance compensation algorithm can be described by [25]:

(6) $$d_{1}=2R\sin^{2}\varphi$$

(7) $$d_{x}=\rho_{r}\cos\theta_{r}$$

(8) $$d_{y}=\left(\rho_{r}\sin\theta_{r}-d_{1}\right)/\cos2\varphi$$

where $d_{x}$ is the longitudinal relative distance between two vehicles, $d_{y}$ is the transverse distance between two vehicles, which is relatively recent, 2φ is the central angle corresponding to the curve, $\rho_{r}$ is the straight-line distance between the connection of the front of ego vehicle and the rear of preceding vehicle, and $\theta_{r}$ is the angle between the velocities of ego and the preceding vehicle.

**Figure 4:**
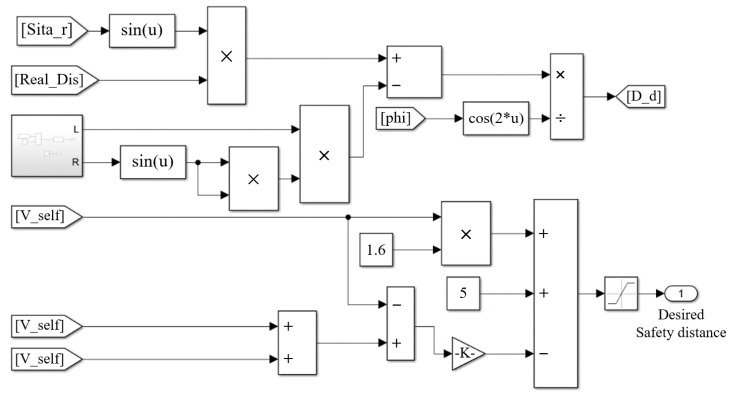
Safety distance model.

The proposed algorithm works by setting a safety threshold to $d_{x}$. Here, the road width is set to 4 m and the threshold is set between −2 and 2 m. Once $d_{x}$ is within the threshold, the adjacent vehicle will be evaluated as an alternative of the following target by $d_{y}$, which in turn would obviate the annoying disturbance. The ultimate model of safety following distance is depicted in Figure 4.

## 3. Desired Acceleration Based on Optimal Control

### 3.1. Optimal Control

Using numerical techniques, the optimal control theory seeks the best result of preset performance indicators from alternative control schemes [26]. The development of computer technology in recent years broadens the possibility of the theory and makes it appealing, particularly with the algorithm of Dynamic Programming (DP) and minimum principle [27]. To address the issue, the motion equations, the value ranges of control variables, the initial and target state of preset motion process, and the performance indicators are essential. The optimal control theory can be restated as solving the extremum of performance indicator function (called functional) with the control function and motion state as variables, under the constraints of motion equations and value ranges. Significantly, the selection of performance indicators largely decides the final effect of the optimal control system.

### 3.2. Desired Acceleration Solution

**Figure 5:**
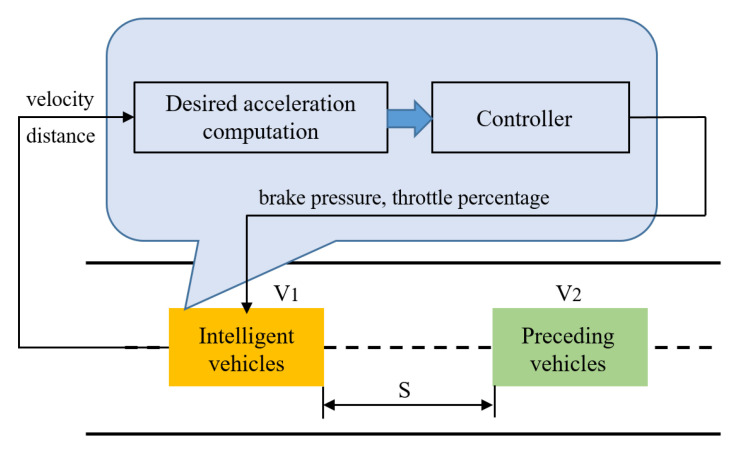
Calculation logic of desired acceleration.

The solution utilizes the differences of velocity and distance between ego vehicle and preceding vehicle as inputs. Based on the relative motion, an objective function is established to solve the desired acceleration for prevention of collision. After receiving the computed result, the controller will output both the brake pressure and throttle percentage in order to compensate the acceleration. The calculation logic is described in Figure 5.

Supposing that the preceding vehicle drives at a constant speed for a short time:

(9) t=dt×i

(10) $$v=v_{1}+at$$

(11) $$s_{1}=v_{1}t+\frac{1}{2}at^{2}$$

(12) $$s_{2}=v_{2}t$$

where *i* is a point in time and dt is the time interval and has the value 0.1 s. $v_{1}$ and $v_{2}$ are the detected velocity of the ego and preceding vehicles, respectively. $s_{1}$ and $s_{2}$ are the distance of the ego and preceding vehicles travelled in time *t*, respectively.

The travel distance at a certain point in the future can therefore be predicted by:

(13) $$s=s_{0}-\Delta s$$

(14) $$\Delta s=s_{1}-s_{2}$$

where *s* is the distance travelled by ego vehicle in future, $s_{0}$ is the initial spacing, and Δs is the distance variation between the ego and preceding vehicles.

**Figure 6:**
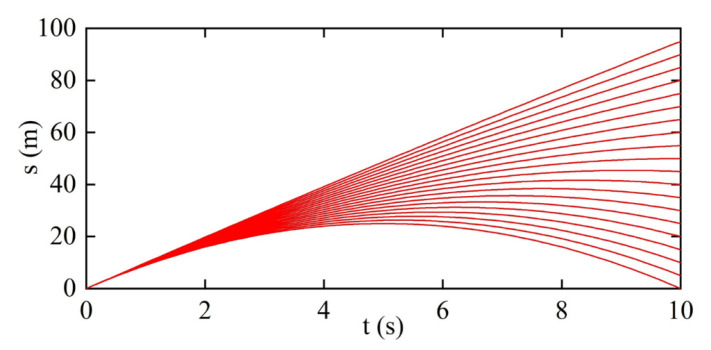
Distance prediction under different acceleration.

Starting from the current moment to the next 10 s, the travel distance of the ego vehicle under different acceleration is predicted in Figure 6. The travel distance of the preceding vehicle can be predicted likewise, using the detected velocity as an approximation for the real velocity in a time interval.

Based on the prediction algorithm, the first part of objective function, considering that the velocity of ego vehicle exerts great influence on acceleration, is set to be the speed cost:

(15) $$J_{1}=k_{v}\sum \frac{1}{v_{1i}^{2}}$$

where $k_{v}$ is the velocity weight and $v_{1i}$ is the velocity of the ego vehicle at time *i*. The second part considers the distance cost. Sigmoid function is introduced as an activation function thanks to its smooth curve and easy derivation, defined by:

(16) $$S\left(x\right)=\frac{1}{1+e^{-x}}$$

**Figure 7:**
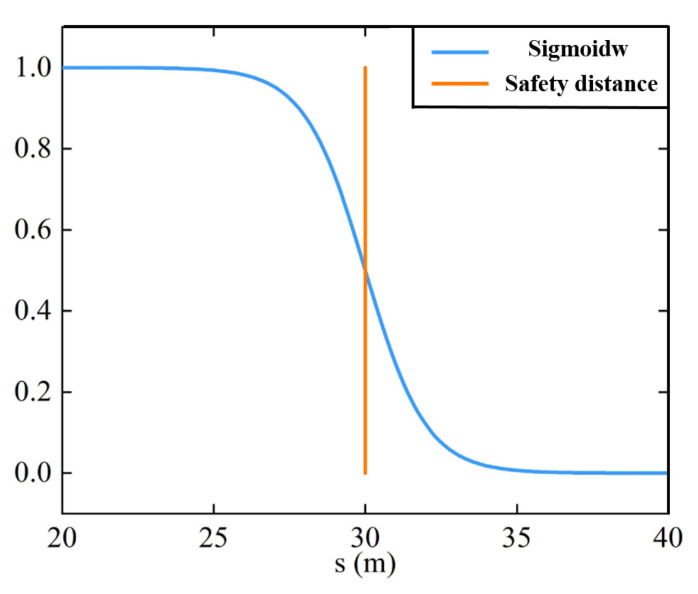
Sigmoid function.

In the context, Equation (16) can also be rewritten as:

(17) $$S_{j}\left(s\right)=1-\frac{1}{1+e^{\left(-n\cdot \left(s-s_{\min}\right)\right)}}$$

where *n* has the value 1, *s* is the distance between ego and preceding vehicles, and $s_{\min}$ is the minimum safety distance set according to different collision-avoidance requirements. The function curve is shown in Figure 7. It shows that the value of the Sigmoid function ranges fleetly from 0 to 1 once $s<s_{\min}$, otherwise it would change rapidly from 1 to 0.

Combining the aforementioned model of safety following distance (Equation (4)) with Equation (17), the second part of the objective function can be given by:

(18) $$J_{2}=k_{s}d_{des}\sum S_{j}\left(s_{i}\right)$$

where $k_{s}$ is the distance weight, $s_{i}$ is the distance between ego and preceding vehicles at time *i*. Sigmoid function will be gradually activated with the decrease of $s_{i}$, after which the value of objective function will be consistent with the trend of $d_{des}$.

Finally, the objective function can be calculated by [28]:

(19) $$J=J_{1}+J_{2}=k_{v}\sum \frac{1}{v_{1i}^{2}}+k_{s}d_{des}\sum S_{j}\left(s_{i}\right)$$

When there is a long distance between ego and preceding vehicles, *J*, as well as the acceleration, have small values. In turn, a large deceleration is required for *J* decrease if spacing distance is rather short or even shorter than the set $s_{\min}$. The performance indicators of the controlled system, by this means, can be perfectly embodied.

**Figure 8:**
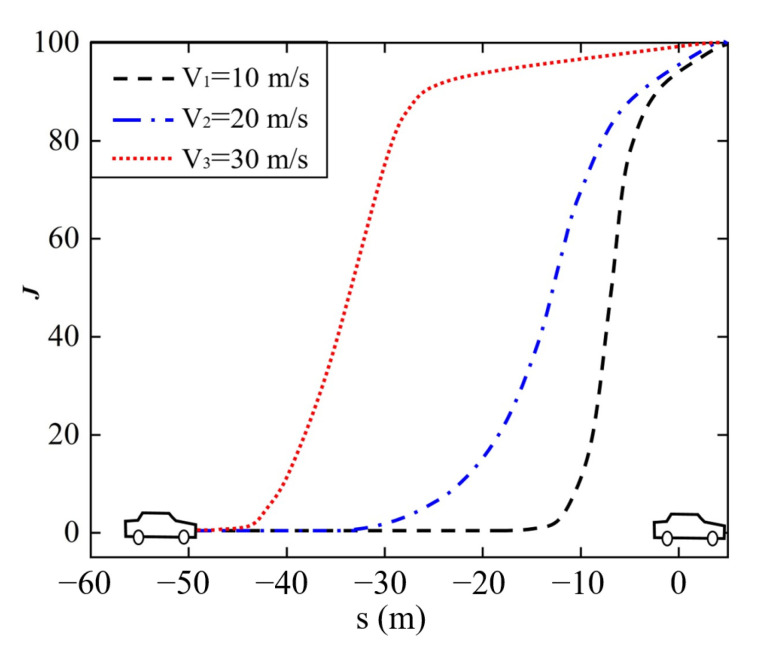
Collision risk prediction.

Significantly, the speed cost $J_{1}$ and distance cost $J_{2}$ jointly depict the collision risk when longitudinally following, as illustrated in Figure 8. It shows that the ego vehicle travels faster than the preceding vehicle and the velocity difference is relatively large; *J* would increase fleetly with the spacing distance getting shorter, ending in a timely deceleration in the ego vehicle. The larger the velocity difference is, under a same spacing distance, the faster *J* grows.

The optimization of objective function results in the desired longitudinal acceleration is provided by longitudinal force and limited by adhesion coefficient on road surface. To enhance the viability of proposed algorithm, several optimization constraints were introduced:

(20) $$s_{i}+d_{s}\leq s_{0}$$

(21) $$v_{\min}\leq v_{f}\leq v_{\max}$$

(22) |a|≤μg

where $v_{\min}=0$, $v_{\max}=1.1v_{des}$, and μ is the road adhesion coefficient. Equations (20)–(22) denote the constraints of following distance, velocity, and acceleration, respectively. With the constraints, the desired acceleration can be well solved with a nonlinear optimization function ‘fmincon’ in the Matlab environment.

## 4. Acceleration Compensation via Longitudinal PID Control

**Figure 9:**
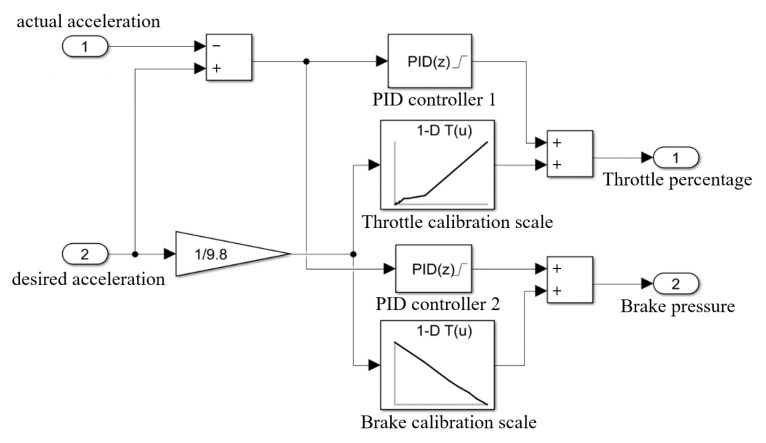
Longitudinal PID control model.

Generally, the desired acceleration is achieved by exerting sufficient brake pressure, which responds rapidly but makes passengers suffer in emergency situations. In this section, the longitudinal PID control method is introduced for acceleration compensation [29]. The brake pressure is combined with throttle percentage for adjustment to improve the ride comfort. The specific strategy and model of the longitudinal PID control are respectively shown in Table 1 and Figure 9. Proportionality coefficient $k_{p}$, integration coefficient $k_{i}$, and derivative coefficient $k_{d}$ are the three control parameters and are respectively assigned after deviation debugging as: $k_{p}=4$, $k_{i}=2$, $k_{d}=0$.

**Figure 10:**
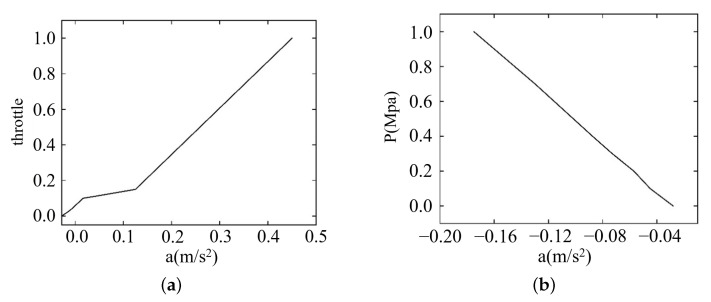
Calibration scale. (**a**) Throttle percentage. (**b**) Brake pressure.

Additionally, in order that the throttle percentage and brake pressure can be consistent with the characteristics of vehicle engine and brake, they are fitted based on the looking-up table, as indicated in Figure 10.

**Figure 11:**
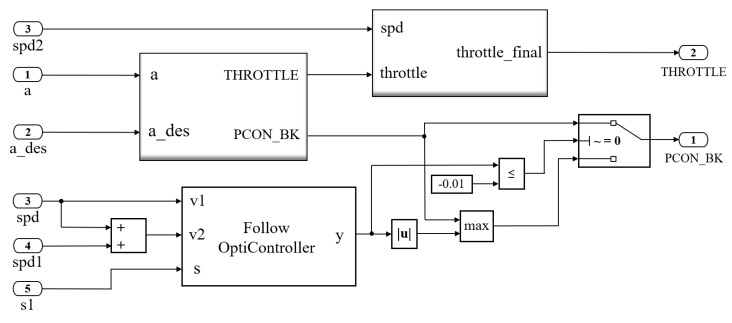
Switching logic of the proposed vehicle-following model.

Combining the longitudinal PID control method with the aforementioned optimal control algorithm, the switching logic of the proposed vehicle-following model is designed, as shown in Figure 11. When collision risks are detected, great brake pressure is conducted while throttle percentage is set to the minimum. Gradually, with the decrease in velocity difference between ego and preceding vehicles, the throttle works in coordination with the brake to raise the stability in the process of vehicle following.

## 5. Simulation Results

Other than some conventional vehicle-following strategies in the ACC system, the devised control system focuses on the requirements of longitudinal collision avoidance. For safety promotion, velocity and distance prediction are embedded to predict if there will be certain collision risks. The performance is examined by different following scenarios in the CarSim/Simulink simulation environment.

### 5.1. Simulation on Straight Roads When the Preceding Vehicle Drives at a Constant Speed

In this part, the velocity of the preceding vehicle is set to be 36 km/h. For the convenience of comparative analysis, the ego vehicle has two initial speed values, i.e., 72 km/h and 108 km/h, respectively. The initial spacing between ego and preceding vehicle is 65 m, and the road adhesion coefficient is 0.9.

**Figure 12:**
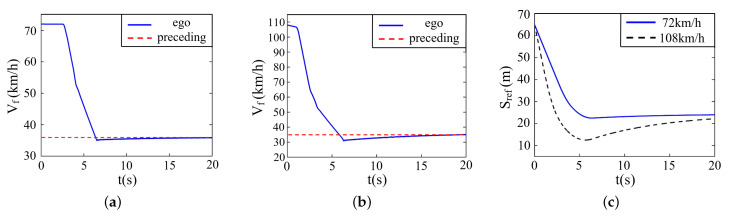
Simulation on straight roads when preceding vehicle drives at a constant speed. (**a**) Velocity (72 km/h). (**b**) Velocity (108 km/h). (**c**) Relative distance.

The simulation result is described in Figure 12, where (a) denotes the velocity variation when the ego vehicle drives at 72 km/h, (b) denotes the velocity variation when the ego vehicle drives at 108 km/h, and (c) denotes the variation of relative distance between two vehicles.

At the beginning, the ego vehicle detects a large velocity difference with the preceding vehicle and conducts a large deceleration. It can be seen from Figure 12 that the velocity of the ego vehicle and the relative distance dwindles quickly and constantly. Once detecting the collision risk, the ego vehicle starts decelerating until achieving the same speed as the preceding vehicle, during which course it runs rather smoothly, and the acceleration is within the safety constraints all along. It should be noted that the proposed control system responds earlier under the initial velocity of 108 km/h compared with 72 km/h, interpreting the validity of prediction algorithms.

### 5.2. Simulation on Straight Roads When the Preceding Vehicle Drives at Various Speeds

**Figure 13:**
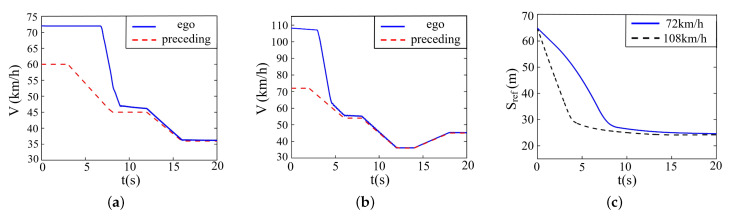
Simulation on straight roads when preceding vehicle drives at various speeds. (**a**) Velocity (72 km/h). (**b**) Velocity (108 km/h). (**c**) Relative distance.

It poses certain risks if there is a sudden change in the velocity of the preceding vehicle. The ego vehicle is supposed to predict the change and respond in advance. Supposing that the preceding vehicle changes speed in two different trends, as shown in subfigure (a) and (b), the simulation result is shown in Figure 13.

It can be seen that the ego vehicle responds rapidly to the first sudden deceleration in the preceding vehicle whether it be driving at 72 km/h or 108 km/h at first. Before the second deceleration, the ego vehicle nearly drives at the same speed with the preceding vehicle and successfully stays in step with it from then on. The larger the initial velocity difference is, the quicker the control system works. Moreover, not limited to the decelerating conditions, the proposed controller tracks perfectly when the preceding vehicle accelerates abruptly, as shown in sub-figure (b). Finally, the relative distance between two vehicles stabilizes at the value of 25 m.

### 5.3. Simulation on Curved Roads When the Preceding Vehicle Drives at a Constant Speed

**Figure 14:**
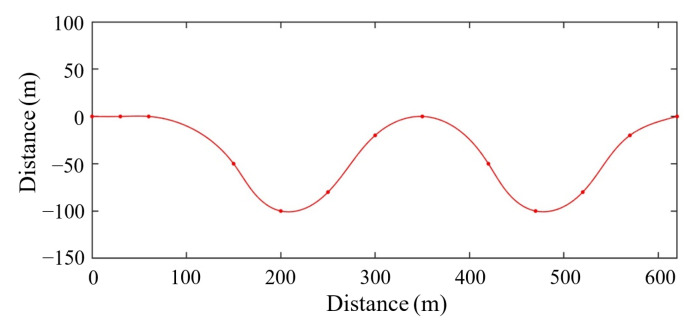
Curved road model.

People usually encounter curved roads in real-world driving conditions. The model of a curved road is established in CarSim, as shown in Figure 14. The road adhesion coefficient is set to be 0.9. The first preceding vehicle (labelled preceding 1) drives at 36 km/h in front of the ego vehicle and has the initial spacing of 65 m. The second preceding vehicle (labelled preceding 2) drives at 25 km/h in the adjacent line and has the initial spacing of 100 m.

**Figure 15:**
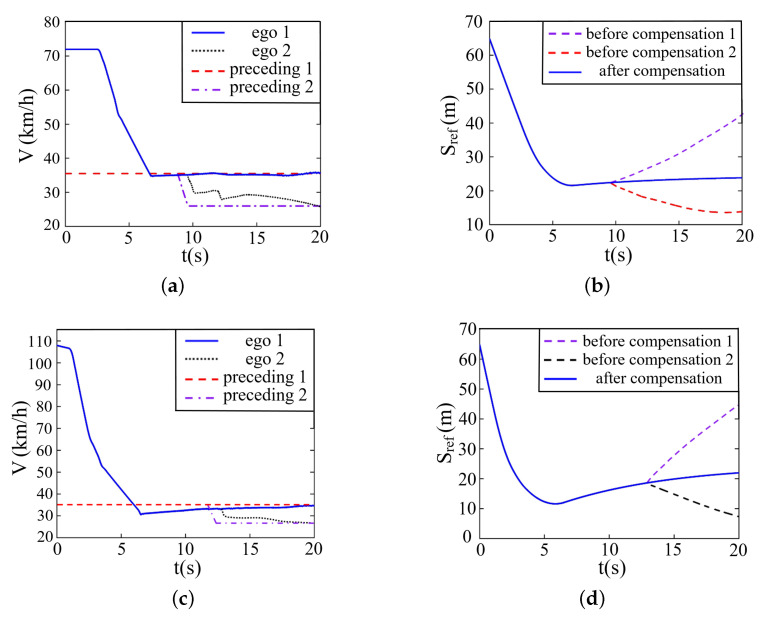
Simulation on curved roads when preceding vehicle drives at a constant speed. (**a**) Velocity (72 km/h). (**b**) Relative distance (72 km/h). (**c**) Velocity (108 km/h). (**d**) Relative distance (108 km/h).

The simulation result is shown in Figure 15. Sub-figures (a) and (b) denote the following conditions with the initial velocity of 72 km/h, and (c) and (d) denote the conditions with the initial velocity of 108 km/h. The ‘ego 1’ marked in sub-figures (a) and (c) represents the velocity variation of the ego vehicle after distance compensation, and the ‘ego 2’ represents the velocity variation before distance compensation. The ‘before compensation 1’ and ‘before compensation 2’ marked in sub-figures (b) and (d) are the relative distance between the ego vehicle and two preceding vehicles, respectively, without distance compensation.

At the beginning, the ego vehicle follows the first preceding vehicle. In a few seconds, the collision risk is detected and the ego vehicle rapidly decelerates to keep the same speed with the first preceding vehicle. It is clear that without distance compensation, when discovering the second preceding vehicle with a shorter spacing and a smaller velocity, the ego vehicle will soon change the following target. The change is totally unnecessary and may be problematic for public transportation. Not to mention, in the subsequent following course, the velocity of the ego vehicle fluctuates on account of the non-linear properties of curved roads, resulting in unpleasant riding experiences. Fortunately, after the distance compensation, the ego vehicle obviates the disturbance of the adjacent vehicle and follows stably after the first preceding vehicle.

### 5.4. Simulation on Curved Roads When Preceding Vehicle Drives at Various Speeds

**Figure 16:**
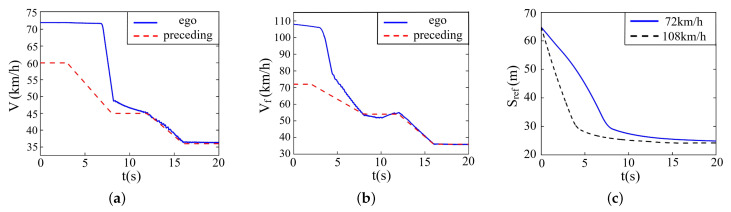
Simulation on curved roads when the preceding vehicle drives at various speeds. (**a**) Velocity (72 km/h). (**b**) Velocity (108 km/h). (**c**) Relative distance.

In this part, the initial velocity of the ego vehicle is set to be 72 km/h and 108 km/h, and the initial velocity of the preceding vehicle is set to be 60 km/h and 72 km/h, respectively. The initial distance between two vehicles is 65 m, and the road adhesion coefficient is 0.9. The preceding vehicle will decelerate twice to the velocity of 36 km/h, and the simulation result is described in Figure 16.

It is noticeable that the proposed controller also puts up a good performance on curved roads. Before the second deceleration occurring in the preceding vehicle, the ego vehicle has already kept up with it. During the whole process, the velocity and relative distance are changing fairly stably. The response time under the condition of a larger velocity difference, which is shorter, enhances the safety performance of the controller.

## 6. Conclusions

With the rapid advancements in autonomous driving, the necessities of the active collision-avoidance system are increasing. This paper proposed a control method for longitudinal vehicle-following based on safety requirements, mainly consisting of a safety distance model, a predictive algorithm, and a control system. The model of safety following distance was depicted based on a fixed time headway. In addition, when devising the spacing policy, the special case on curved roads was typically discussed. A distance compensation algorithm was proposed for the ego vehicle to determine whether to change the following target or not. Later the velocity and distance prediction algorithm quantized the collision risks, based on which an objective function was established to solve the desired acceleration using optimal control theory. Two parts were contained in the objective function, i.e., the speed cost and the distance cost, which highly improved the performance of prediction and safety. The control system was devised using the method of longitudinal proportion integration differentiation to track the desired acceleration, with the outputs of throttle percentage and brake pressure. Ultimately, the effectiveness and validity of the proposed control method was demonstrated under four typical running conditions in the co-simulation of CarSim/Simulink.

In the future work, the lateral control would be included in collision avoidance to cover more dynamic driving conditions. The objective function may contain more performance indicators, such as economy, comfort, emission, etc., in order to satisfy the requirement of comprehensive performance.

## Figures and Tables

**Table 1:** Longitudinal control strategy.

PID Closed-Loop Control of Vehicle Velocity		Open-Loop Control of Calibration Scale	
Input	current acceleration deviation	Input	acceleration
Output	acceleration compensation	Output	throttle percentage/brake pressure

## Data Availability

Not applicable.
